# Analgesic mechanism of electroacupuncture in a rat L5 spinal nerve ligation model

**DOI:** 10.3892/etm.2015.2165

**Published:** 2015-01-02

**Authors:** CHUNCHUN XUE, LEI XIE, XIA LI, JIANFENG CAI, ZHEN GU, KAIQIANG WANG

**Affiliations:** 1 Pain Management Center, Shanghai Hospital of Traditional Chinese Medicine, Shanghai University of Traditional Chinese Medicine, Shanghai 200071, P.R. China; 2 Department of Orthopaedics and Traumatology, Jiashan Hospital of Traditional Chinese Medicine, Jiashan, Zhejiang 314100, P.R. China

**Keywords:** electroacupuncture, spine nerve ligation, brain-derived neurotrophic factor, purinergic receptor P2X, ligand-gated ion channel 4

## Abstract

The aim of this study was to investigate the analgesic mechanism of electroacupuncture (EA) in the treatment of neuropathological pain. A total of 60 Sprague-Dawley rats were randomly divided into three groups, namely the spinal nerve ligation (SNL), electroacupuncture (SNL + EA) and normal control groups, with 20 rats in each group. The up-down method was used to determine the bipedal 50% mechanical paw withdrawal threshold (PWT). The ultrastructure of the injured-side L5 nerve root (n=6) was observed by electron microscopy. The mRNA levels of brain-derived neurotrophic factor (BDNF) and purinergic receptor P2X, ligand-gated ion channel 4 (P2X4) in the spinal cord (n=14) were determined by reverse transcription-quantitative polymerase chain reaction (RT-qPCR). The postoperative PWT of the injured-side hindpaw in the SNL group at each time point was lower than that in the control group (P<0.01); there were differences of statistical significance between the PWT values of the SNL + EA and SNL groups on postoperative days 14 and 21 (P<0.05). Postoperatively, the PWT of the hindpaw on the uninjured-side was significantly lower in the SNL group when compared with that of the control group on days 10, 14 and 21 (P<0.05). Following the EA treatment, axonal demyelination was reduced and vascular proliferation was observed within the visual field. In addition, following the EA treatment, BDNF expression levels in the spinal dorsal horn increased (P<0.05), while the expression of P2X4 was not different from that in the SNL group. EA exerted an analgesic effect on the SNL model in a time-dependent manner, and improved the blood supply to the nerve root. Following the EA treatment, the expression of P2X4 did not change significantly compared with that in the SNL group, whereas the spinal secretion of BDNF increased. However, the exact mechanism requires further study.

## Introduction

The spinal nerve ligation model (SNL) was first proposed by Kim and Chung in 1992 (1). Since the manifestations of neuropathological pain (NP), such as spontaneous pain, mechanical allodynia and thermal hyperalgesia, can be quickly induced postoperatively, and the pain maintenance time is long (1), SNL has become the most commonly used model of NP. In recent years, animal experiments and clinical research studies have shown that electroacupuncture (EA) treatment has a clear analgesic effect towards NP (2,3). Studies have shown that when NP occurs, the spinal levels of purinergic receptor P2X, ligand-gated ion channel 4 (P2X4) and microglial levels of brain-derived neurotrophic factor (BDNF) play important roles in the generation and maintenance of NP (4,5); however, it remains unclear whether EA affects the spinal expression of P2X4 and BDNF in SNL model rats during analgesia.

In the present study, the up-down method was used to measure the bipedal 50% mechanical paw withdrawal threshold (PWT), electron microscopy was used to observe the ultrastructure of the injured-side L5 nerve root (n=6) and reverse transcription-quantitative polymerase chain reaction (RT-qPCR) was performed to detect the mRNA levels of BDNF and P2X4 in the spinal cord. The aim of the study was to investigate the relationship between analgesia and repair, and thus provide a theoretical basis for the mechanism of EA-induced analgesia.

## Materials and methods

### Experimental animals

A total of 60 male Sprague-Dawley (SD) rats, weighing 220–250 g, were provided by the Animal Center of Shanghai SLAC Laboratory Animal Co., Ltd. (Shanghai, China). The animals were then reared in separated cages in the Animal Center of the Affiliated Municipal Hospital of Traditional Chinese Medicine (TCM), Shanghai University of TCM (Shanghai, China). The room conditions were maintained at 23±2°C and 50–60% humidity, with day-night cycling lighting (12 h-12 h). All experimental steps were performed with the purpose of minimizing the suffering of the animals and operated in accordance with the relevant principles of laboratory animal care. The experimental animals were reared in the above environment for a week for adaption prior to the experiment. This study was carried out in strict accordance with the recommendations in the Guide for the Care and Use of Laboratory Animals of the National Institutes of Health (8th edition, 2011). The animal use protocol was reviewed and approved by the Institutional Animal Care and Use Committee (IACUC) of Shanghai Hospital of TCM, Shanghai University of TCM.

### Preparation of the SNL model and animal grouping

The animals were satisfactorily anesthetized with 10% chloral hydrate (300 mg/kg) and the fur was removed by shaving. The rats were then fixed, disinfected and laid on a towel. The L5/6 spinous gap was positioned in the center, and an incision was made ~4 cm along the midline of the back. The skin and subcutaneous tissues were cut and the paraspinal muscles were bluntly dissected and fixed with a retractor. Following blunt removal of the left L5 lamina and zygopophysis to expose the left L5 nerve root and dorsal root ganglia, a 5-0 Mersilk thread was used to ligate the L5 nerve tightly. The wound was then washed with saline to fully stop the bleeding, and a 4-0 Vicryl absorbable suture was used to suture the muscle fascia, subcutaneous tissue and skin layer by layer. Gentamicin was then administered by intramuscular injection to prevent infection. The rats in the normal control group were not subjected to surgery. All the animals were kept in individual cages, with room temperature maintained at 23±2°C, natural lighting and free access to water and food.

The 60 SD rats were randomly divided into three groups, namely the spinal nerve ligation group (SNL group, n=20), the normal control group (control group, n=20) and the electroacupuncture group (SNL + EA group, n=20). The SNL group and the control group were not given any treatment.

### Determination of bipedal 50% mechanical PWT

The behavioral test time was fixed at 8:00–12:00, and the testers did not know the surgery grouping. The rat bipedal pain thresholds were determined on preoperative day 1 and postoperative days 1, 3, 5, 7, 10, 14 and 21. The rat was placed in a transparent Plexiglass box, the bottom of which was a 5×5 mm wire mesh and allowed to adapt for ~15 min. A series of standardized von Frey fibers (Stoelting Co., Ltd., Wood Dale, IL, USA) was then used to stimulate the center of the rat hindpaw, with a stimulating force that caused the fiber to form a slight S-shape and continued for 6–8 sec. When the rat had calmed down, the mechanical pain threshold was measured according to the up and down method described by Dixon (6). The folding forces of the classical von Frey fibers were 0.4, 1.4, 2.0, 4.0, 6.0, 8.0 and 15.0 g. Started from 2.0 g, the von Frey fiber was vertically pushed into the plantar skin of the rat hindpaw, with sufficient force to cause the fiber to bend into an S-shape, taking care to avoid the paw pad. Then, dependent upon the paw withdrawal, the fiber was replaced with the next or previous fiber, with the testing of each fiber lasting no longer than 8 sec, and the interval between adjacent tests being 10 sec. The reactions of the rats towards the different fibers in the series were recorded. If the rat quickly flinched or licked the paw, the reaction was recorded as positive, expressed as X; if there was no response, then the reaction was considered as negative, expressed as O. A series of Os or an O and X combination sequence was then be obtained; an O anterior to X was set as the starting point, and six consecutive stimuli, including the starting point, such as OXOXOO, were used as the key sequence for the estimation of the 50% mechanical PWT. The estimation formula was as follows: 50% mechanical PWT (g) = (10[Xf+κδ])/10,000, where Xf is the logarithmic value of the last von Frey fiber in the sequence; κ is the value obtained by table search according to the measured X and O sequence; and δ is the logarithmic average difference of each fiber intensity, which in this study was ~0.224. The estimated 50% PWT value by this formula may be >15.0 g or <0.4 g, while 15.0 and 0.4 g continued to be used as the maximum and minimum mechanical pain thresholds. If the stimuli in the force range 2.0–0.4 g were all positive, the 50% PWT was recorded as 0.4 g; if the stimuli in the force range 2.0–15.0 g were all negative, the 50% PWT was recorded as 15.0 g. Measurement of the mechanical pain threshold of the uninjured side was conducted 15 min after that of the injured side. If the rat appeared to have a preoperative right paw 50% mechanical PWT ≤4.0 g, the rat was excluded from the study. The rats were placed into the experimental observation box for 30 min/day one week prior to the experiment for adaption.

### EA intervention

Seven days after modeling, the rats were satisfactorily anesthetized, then fixed on a special board. The acupoints of ‘Huantiao’ and ‘Zusanli’ in the injured side were electrically stimulated with the BT701-1B EA device (Shanghai Huayi Medical Instrument Co., Ltd., Shanghai, China) using the following EA parameters: frequency, 2 Hz; current intensity, ≤1 mA (a slight tremble in the left lower limb muscles was set as the limit). Stimulation for 30 min/day for 7 days was a course of treatment and two consecutive courses were conducted. The SNL + A group received the EA treatment, while the rats in the other two groups were anesthetized, but did not receive the EA treatment.

### Sampling

All rats were intraperitoneally injected with 10% chloral hydrate (300 mg/kg) for satisfactory deep anesthesia on postoperative day 21. Twelve rats in each group were randomly selected and sacrificed by decapitation. The L5-6 section of the spinal cord was quickly removed, placed on ice, and put into a 200-μl RNAiso Plus EP tube, then frozen in liquid nitrogen for RT-qPCR analysis. Two samples from each vertebral point of the spinal cord were taken to form a PCR sample.

### RT-qPCR

Total RNA was extracted from spinal cord samples using TRIzol reagent (Invitrogen Life Technologies, Carlsbad, CA, USA). RT was performed using an RT-for-PCR kit (Qiagen, Valencia, CA, USA) following the manufacturer’s instructions. The primers were designed and synthesized by Shanghai Biological Engineering Technology & Services Co., Ltd. BDNF primer sequences: 5′-CAGTGGCTGGCTCTCATACC-3′ and 3′-CGGAAACAGAACGAACAGAA-5′; P2X4 primer sequences: 5′-CGTGGCGGACTATGTGATT-3′ and 3′-GGTGCTCTGTGTCTGGTTCA-5′; the internal reference was GAPDH. RT-qPCR amplifications were performed using the SYBR Premix Ex Taq kit (Takara Bio, Dalian, China) in an RG 3000 thermal cycler (Corbett Research, Qiagen) with 4 μl sample per assay. The qPCR conditions were as follows: holding stage, 95°C for 10 min; and cycling stage, 95°C for 15 sec, 60°C for 1 min and 72°C for 35 sec, followed by melt curve analysis to confirm the specificity of the amplified products. The fold change was calculated by the 2^−ΔΔCT^ method. PCR products were subjected to melting curve analysis, while the data were quantified using RotorGene 6.0 analysis software (Qiagen). The experiments were repeated at least three times.

### Electron microscopy

The remaining six rats of each group were subjected to perfusion-fixation, and then the ligated nerve root of the injured-side L5, approximately the size of a grain of rice, was immersed in a 2.5% glutaraldehyde-prepared EP tube (Sigma-Aldrich, St. Louis, MO, USA) for observation under a CM120 electron microscope (Philips, Zürich, Switzerland).

### Statistical analysis

SPSS statistical software package, version 18.0 (SPSS, Inc., Chicago, IL, USA) was used for the statistical analysis, The measurement data are expressed as the mean ± standard deviation, the differences among the pain thresholds of the repeated measurements in each group were subjected to analysis of variance (ANOVA) with repeated measurement data, intergroup comparisons at the same time point were conducted using ANOVA, with P<0.05 considered to indicate a statistically significant difference.

## Results

### 50% mechanical PWT results

**Figure 1:**
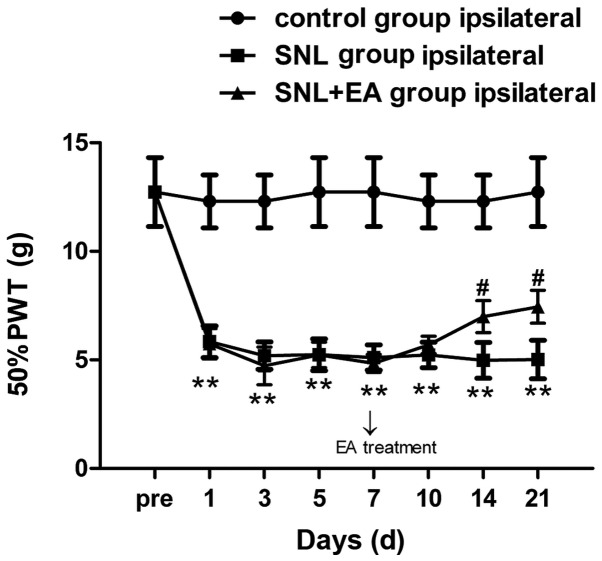
Changes of 50% PWT in the injured (ipsilateral) side of rats of each group following EA treatment. ^**^P<0.01, compared with the ipsilateral control group; ^#^P<0.05, compared with the ipsilateral SNL group. PWT, paw withdrawal threshold; EA, electroacupuncture; SNL, spinal nerve ligation.

**Figure 2:**
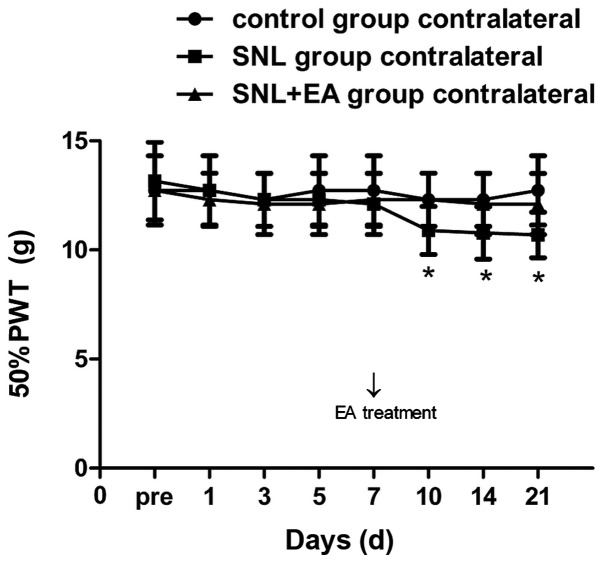
Changes of 50% PWT in the uninjured (contralateral) side of rats of each group following EA treatment. ^*^P<0.05, compared with the contralateral control group. PWT, paw withdrawal threshold; EA, electroacupuncture; SNL, spinal cord ligation.

There was no significant difference among the preoperative bipedal 50% PWT values in the different groups. Compared with the preoperative baseline value, the injured-side 50% PWT in the SNL group decreased significantly on postoperative day 1, and was maintained at a lower level from postoperative day 7 to the end of the observation period and was significantly different compared with that in the control group (P<0.01). Compared with the control group, the mechanical pain threshold in the SNL + EA group increased slightly (P>0.05). The increase in the mechanical pain threshold in the SNL + EA group compared with that in the SNL group was of statistical significance only on postoperative days 14 and 21 (P<0.05), while no statistical significance was identified at the other time points (P>0.05; Fig. 1). The 50% PWT of the uninjured-side hindpaw in the SNL group was decreased compared with that of the control group from postoperative day 10, exhibiting a significant difference on postoperative days 10, 14 and 21 (P<0.05); no significant differences were identified at the other time points. The 50% PWT of the uninjured-side hindpaw in the SNL + EA group was significantly different compared with that in the SNL group on days 10, 14 and 21 (P<0.05; Fig. 2).

### Electron microscopy results

**Figure 3:**
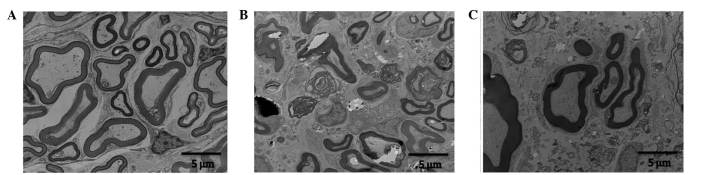
Ultra-microstructure of the ipsilateral L5 spinal nerve in each group. (A) Normal control group; (B) SNL group; (C) SNL + EA group. SNL, spinal nerve ligation; EA, electroacupuncture.

In the control group, the myelin structure of the nerve root was integrated, the axonal structure was normal and arranged regularly, and the Schwann cells were normal. In the SNL group, the majority of the myelin structure of the nerve root had disappeared, axons were bare and arranged irregularly, while the axonal structure was integrated, Schwann cells proliferated and myelin debris was visible in the cytoplasm, with largely proliferated Schwann cell nuclei. In the SNL + EA group, the axons of the nerve root were partially demyelinated, the majority of the axonal myelin was complete but thinner, the axonal structure was integrated although arranged irregularly, the proliferation of Schwann cells was not evident and vascular proliferation was observable within the field of vision (Fig. 3).

### Expression levels of BDNF mRNA

**Figure 4:**
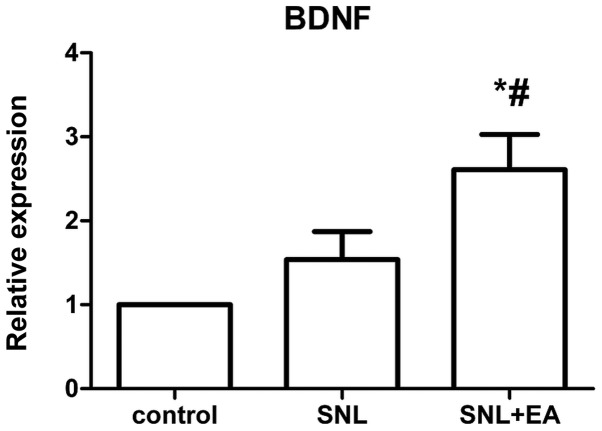
Changes in the expression of BDNF mRNA in the spinal horn of rats following EA treatment. ^*^P<0.05, compared with the control group; ^#^P<0.05, compared with the SNL group. BDNF, brain-derived neurotrophic factor; SNL, spinal nerve ligation; EA, electroacupuncture.

The expression level of BDNF mRNA in the SNL + EA group was significantly higher compared with that in the SNL group (P<0.05), and the expression of BDNF mRNA in the SNL group was not significantly different compared with that in the control group (P>0.05; Fig. 4).

### Relative expression of P2X4 mRNA

**Figure 5:**
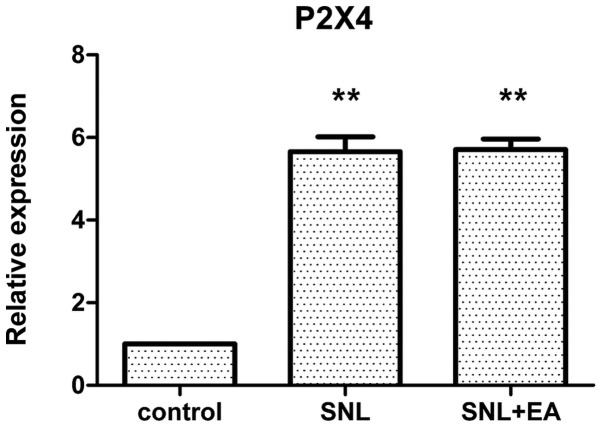
Changes of expression of P2X4 in the spinal horn of rats following EA treatment. ^**^P<0.01, compared with the control group. P2X4, purinergic receptor P2X, ligand-gated ion channel 4; SNL, spinal nerve ligation; EA, electroacupuncture.

No significant difference was identified in the expression levels of P2X4 mRNA between the SNL + EA and the SNL groups (P>0.05). The P2X4 mRNA expression levels in the SNL and SNL +EA groups were significantly higher than that in the control group (P<0.01; Fig. 5).

## Discussion

The results of this study demonstrated that compared with the preoperative baseline value, the postoperative 50% PWT in the SNL group decreased progressively, and the hyperalgesia was significant, indicating that the SNL modeling was successfully established. In addition, the mechanical hyperalgesia was present on postoperative day 1 in the SNL group, and was significantly decreased on postoperative day 3, reaching its lowest level on postoperative day 7 and maintaining a low level until the end of the observation period; the results were consistent with those of previous studies (1). Compared with the preoperative state, hyperalgesia of the uninjured-side hindpaw was statistically significant on postoperative days 10–21 (P<0.05), the so-called ‘mirror pain’ phenomenon, which is consistent with the results of Arguis *et al* (7). Mirror pain refers to the concept that when a peripheral nerve is injured, pain can be perceived not only from the injured area, but also from sites a certain distance outside the injured area (8). Sometimes the pain occurs in the contralateral side, and the contralateral pain is similar in nature to the ipsilateral pain; while not significant, the mirror pain phenomenon of the animal model is often ignored in studies (9). Certain patients with complex regional pain syndrome (CRPS) or post-herpetic neuralgia also appear to experience mirror pain (10,11); however, the mechanism remains unclear, with current hypotheses focusing on neural network and immune activation theories (12). The immune activation theory considers that when the peripheral nerves are injured, a large number of metabolites are produced at the injured site, and a large number of immune cells infiltrate; the damage-induced metabolites and the immune response may be associated with the activation of spinal glial cells and the release of inflammatory mediators (13,14). However, the immune theory cannot explain the presence of allodynia in the bilateral corresponding regions. As the nervous system has anatomical symmetry, peripheral and central neural pathways are likely to involve in the process of mirror pain. In addition, the study of anatomy has determined that symmetric neural connections occur at the level of the spinal cord (15). The exact reaction site characteristics on the opposite side could only be completed through a neural pathway. Therefore, in the current study, it is hypothesized that the only immune factor is a pain signaling molecule, and that immune factors, associated with the specific neural pathways, perform the functions together. A recent study (16) reported that in the chronic constriction injury model, enhanced MRI revealed that when a unilateral nerve was injured, activities were exhibited by nerves bilaterally. Therefore, studies of mirror pain are required to not only investigate mirror pain itself, but also to determine the nature of the pain, which may improve the understanding of how the two sides of the body are connected together.

The RT-qPCR results demonstrated that the BDNF mRNA expression level in the SNL + EA group was higher compared with that in the SNL group, with statistical significance (P<0.05), while the 50% PWT in the SNL + EA group was also higher than that in the SNL group. The combination of these two results shows that EA induces the body to secrete BDNF, and thus exhibits analgesic effects. The increase in the pain threshold was positively correlated with the EA treatment time. The level of BDNF mRNA expression in the SNL group was increased slightly compared with that in the control group, without statistical significance, while the pain threshold was significantly lower than that in the control group. A previous study (17) found that in the EA-non-intervention SNL model, quantitative ELISA analysis confirmed that BDNF appears with a secretion peak in the early stage of injury (within 24–48 h), and returns to the preoperative control level in 28 days. In the present study, it was found that following EA intervention, the BDNF content in the spinal dorsal horn significantly increased on postoperative day 21, indicating that repeated EA treatment continued to promote BDNF secretion. Other studies (18,19) have shown that when a nerve is injured, ATP release from the central process endings of spinal dorsal horn neurons is increased, which activates the P2X4 receptor on the microglia of the spinal dorsal horn, leading to the opening of nerve cell membrane Ca^2+^ channels, and thereby promoting the release of BDNF. The released BDNF then binds to its receptor trkB, regulating the phosphorylation and decreasing the activity of the K^+^-Cl^–^ co-transporter 2 (KCC2), causing dysfunction of inhibitory neurons, and thus generating pain; that is, pain is induced by the P2X4R-BDNF-trkB-KCC2 pathway in microglial cells. The present study observed that in the SNL group the P2X4 content of the spinal dorsal horn increased significantly compared with that in the control group, with statistical significance (P<0.05), while compared with the SNL + EA group, the P2X4 content showed no significant differences. Therefore, it may be presumed that the analgesic mechanism of EA was not associated with changes in the expression of P2X4.

Electron microscopy results revealed that after modeling, the axons of the ligated nerve root were irregular, Schwann cells proliferated, and myelin debris was visible in the cytoplasm. A large number of proliferated Schwann cell nuclei were observed and the number of endoplasmic reticulums increased. In addition, the axons were irregularly arranged, and medullary droplets were present, all of which are consistent with the characteristics of Wallerian degeneration following injury of the peripheral nerve. However, following EA treatment, axonal demyelination in the SNL + EA group was reduced compared with that in the SNL group, Schwann cell proliferation was not evident, and vascular proliferation was visible in the field of vision. Therefore, it may be concluded that EA reduced the mechanical damage-induced demyelination of nerve roots, promoted the vascular proliferation of the nerve root, improved blood supply to the nerve root and promoted neurological recovery. This result is consistent with a previous study (20); however, the exact mechanism remains unclear. The effect of EA may be associated with the formation of a stable electric field that may promote injured nerve regeneration (21), or with the rhythmic contractions of stimuli-involved muscles, which may promote local blood circulation (22).
